# Moral distress and ethical climate in intensive care medicine during COVID-19: a nationwide study

**DOI:** 10.1186/s12910-021-00641-3

**Published:** 2021-06-17

**Authors:** Moniek A. Donkers, Vincent J. H. S. Gilissen, Math J. J. M. Candel, Nathalie M. van Dijk, Hans Kling, Ruth Heijnen-Panis, Elien Pragt, Iwan van der Horst, Sebastiaan A. Pronk, Walther N. K. A. van Mook

**Affiliations:** 1 Department of Intensive Care, Maastricht University Medical Center+, PO Box 5800, 6202 AZ Maastricht, The Netherlands; 2 Department of Methodology and Statistics, Care and Public Health Research Institute (CAPHRI), Maastricht University, Maastricht, The Netherlands; 3 Department of Spiritual Care Services, Maastricht University Medical Center+, Maastricht, The Netherlands; 4 Academy for Postgraduate Medical Training, Maastricht University Medical Center+, Maastricht, The Netherlands; 5 School of Health Professions Education, Maastricht University, Maastricht, The Netherlands

**Keywords:** Moral distress, Ethical climate, Intensive care medicine, Nurses, Intensivists, Doctors, COVID-19, Coronavirus

## Abstract

**Background:**

The COVID-19 pandemic has created ethical challenges for intensive care unit (ICU) professionals, potentially causing moral distress. This study explored the levels and causes of moral distress and the ethical climate in Dutch ICUs during COVID-19.

**Methods:**

An extended version of the Measurement of Moral Distress for Healthcare Professionals (MMD-HP) and Ethical Decision Making Climate Questionnaire (EDMCQ) were online distributed among all 84 ICUs. Moral distress scores in nurses and intensivists were compared with the historical control group one year before COVID-19.

**Results:**

Three hundred forty-five nurses (70.7%), 40 intensivists (8.2%), and 103 supporting staff (21.1%) completed the survey. Moral distress levels were higher for nurses than supporting staff. Moral distress levels in intensivists did not differ significantly from those of nurses and supporting staff. “Inadequate emotional support for patients and their families” was the highest-ranked cause of moral distress for all groups of professionals. Of all factors, all professions rated the ethical climate most positively regarding the culture of mutual respect,  ethical awareness and support. “Culture of not avoiding end-of-life-decisions” and “Self-reflective and empowering leadership” received the lowest mean scores. Moral distress scores during COVID-19 were significantly lower for ICU nurses (*p* < 0.001) and intensivists (*p* < 0.05) compared to one year prior.

**Conclusion:**

Levels and causes of moral distress vary between ICU professionals and differ from the historical control group. Targeted interventions that address moral distress during a crisis are desirable to improve the mental health and retention of ICU professionals and the quality of patient care.

**Supplementary Information:**

The online version contains supplementary material available at 10.1186/s12910-021-00641-3.

## Introduction

During a global health crisis, e.g. the COVID-19 pandemic, caregivers encounter ethical issues they usually do not experience in everyday healthcare [1, 2]. These issues can be summarized in what Wynia refers to as the three “Rs”: rationing, restrictions, and responsibilities [3]. Health care professionals have to allocate limited resources fairly, whether they are medical (e.g., drugs, ventilators, testing capability, and hospital beds), structural (e.g., venue) or human (e.g., medical personnel) [4]. Restrictions such as isolation and quarantining of patients, their families and health care professionals may additionally affect the delivery of health care [5]. Furthermore, healthcare professionals are held responsible for the quality of care for their patients despite their personal risks. As a consequence, these professionals have to balance their own and their loved ones’ physical and mental health needs with those of their patients.

As the need for intensive care treatment and support has rapidly increased during the COVID-19 pandemic, the ethical issues described above may apply in particular to the intensive care unit (ICU) professionals. This may cause some to experience so-called “moral distress”.

The term moral distress is defined as knowing *what to do* in an ethical situation, but being *constrained* in some way *from doing* this [6]. An important contributor that either alleviates or aggravates moral distress in health care professionals is the ethical climate in their units and organization. Several researchers in health care settings identified that the more positive the ethical climate is perceived, the less moral distress is reported [7–9].

Studies examining moral distress and ethical climate in healthcare professionals have found that both are associated with the intention to leave one’s job [10–20]. In 2019, a questionnaire study in 84 intensive care units in the Netherlands already revealed that nurses and intensivists experience high levels of moral distress in daily clinical practice (14.9% and 7.5% respectively) [21]. This may be compounded during the COVID-19 pandemic. Experience with previous epidemic health emergencies has shown that distress during an outbreak can cause long-term psychological effects on healthcare professionals such as anxiety, post-traumatic stress disorder, and depression [22–28].

Several studies have investigated the mental health state and factors contributing to burnout of health care professionals exposed to the COVID-19 disease [29–34]. Recently, a study conducted among ICU professionals in a Dutch university medical center and teaching hospital identified three issues that became significantly more distressing during COVID-19 compared to one year prior: scarcity of resources, time, and/or staff, and having to work with colleagues believed insufficiently skilled and/or work unsafely. Despite their interesting findings, a national study is so far lacking [35].

Consequently, this study aims to assess levels of moral distress and quality of ethical climate experienced by intensive care professionals in the Netherlands during the COVID-19 pandemic and to determine factors that cause moral distress in these health care professionals. Furthermore, this study aims to compare the degree of moral distress *during* the COVID-19 outbreak with those of a cohort one year before the COVID-19 outbreak.

## Method

This study was performed between April and June 2020, among all 84 ICUs in the Netherlands. This was just after the first, and so far largest peak of 1320 COVID-19 (suspected) patients admitted to the ICU in the Netherlands on April 7th. By June 11th, the end of the study period, this number had been reduced to 115 patients [36].

### Participants

All ICU team managers were asked to distribute an online questionnaire by email to intensivists, ICU-nurses, and supporting staff (all personnel from other departments called upon, but not primarily trained to deliver ICU care) working in the ICU during the COVID-19 pandemic. Intensivists and ICU nurses were also approached by email through their representative organizations (the Dutch Association for Intensive Care and the Dutch Intensive Care Association for Nurses & Healthcare Assistants respectively). At the time of the study, 3800 ICU-nurses and 826 intensivists were working in the Dutch ICUs, with 63.8% male and 36.2% female intensivists. The number of supporting staff deployed in the Dutch ICUs was unknown. For intensivists, the age distribution was as follows: 30–39 years: 12.5%; 40–49 years: 44.9%; 50–59 years: 31.7% and 60–69 years: 10.9%. The age and gender distribution was unknown for ICU nurses.

### Ethical approval, informed consent, data analysis

This study was approved by the Central COVID-19 Research Committee and the Board of Directors of Maastricht University Medical Centre. Participation was strictly voluntary, and no incentives were offered. Participants were provided an information letter and were only able to participate after providing written informed consent. Data were collected via an online survey software, Qualtrics [37], and were securely stored and analysed according to the General Data Protection Regulation. Data could not be traced back to specific participants.

### Questionnaire

The questionnaire included the Measure of Moral Distress for Healthcare Professionals (MMD-HP) [38], the ethical decision-making climate questionnaire (EDMCQ) [39], and demographic questions. Twenty items that specifically related to the COVID-19 pandemic were added to the questionnaire. A copy of the questionnaire is provided in Additional file 1.

#### MMD-HP

The MMD-HP consists of 27 different clinical situations and an option to suggest other clinical situations of moral distress. Each item was rated on a five-point Likert scale for the frequency of occurrence (0 = never; 4 = very frequently) and the level of distress (0 = none; 4 = very distressing). The moral distress score of each item resulted from multiplying frequency of occurrence and level of distress (range 0–16). After every page break, a comment field was added to allow respondents to clarify their responses. The MMD-HP furthermore contains questions about resignation due to moral distress, providing services that address moral distress and suggestions on how to reduce moral distress. The MMD-HP was back and forth translated from English to Dutch.

#### EDMCQ

The EDMCQ consists of 32 items to assess the ethical climate in the ICU context in the domains of team climate, leadership by physicians, and end-of-life (EOL) care. The original Dutch version of the EDMCQ was used. Each item was scored on a five-point Likert scale from *strongly disagree* to *strongly agree*. A comment field was added after each domain.

#### Development of COVID-19 related items

The previously validated MMD-HP was extended by adding eight items regarding specific COVID-19 aspects, such as family and patient support and contamination fear. Also, twelve items were added to the original EMDCQ questionnaire. To compile these extra questions, an initial pool of questionnaire items was generated based on the available literature [40–43]. Relevance and possible difficulties of comprehensibility or interpretation of the added items were discussed in an expert panel consisting of ICU-professionals. Items were adjusted or discarded if deemed necessary. The questionnaire was subsequently distributed among the participants, no pilot study was performed. However, Cronbach’s alpha calculated for the items added to the MMD-HP and EDMCQ evidenced a rather high internal consistency (0.85 and 0.87 respectively). Exploratory factor analysis (EFA) of the extended MMD-HP and extended EDMCQ was performed (see *analysis*). Additional factors with high internal consistencies emerged from the extra items, indicating an added value of the extra items. See Additional files 2 and 3.

### Analysis

#### Quantitative analysis

Quantitative data were analysed using SPSS v25.0.0.1 [44]. The chi-square goodness-of-fit-test was conducted to assess whether the respondents are representative for the entire population of ICU professionals. The distribution of age and gender of the intensivist group was compared with the numbers expected based on the national distribution. A non-response bias analysis for ICU nurses and supporting staff could not be performed due to the lack of available national data.

Exploratory factor analysis (EFA) was performed to identify the underlying constructs between item scores in the extended MMD-HP and EDMCQ, and thus to check the construct validity of the scales. Principal axis factoring was conducted on the 35 items with oblique rotation. The number of factors to be retained was determined by using the Kaiser Criterion and the scree plot. The factors were interpreted by examining the pattern matrix. Only those items correlating 0.30 or higher with a factor in the rotated solution were considered to be associated with that factor. The score for each factor was calculated by summing the item scores (frequency times level of distress) of the items associated with a factor and dividing this sum by the number of items per factor. EFA of the extended EDMCQ was conducted in the same manner, as well as the calculation of the factor scores, except that no multiplication was needed to obtain the item scores. The identified factors and factor loadings per item are provided in Additional file 2. The internal consistency of the sets of items associated with the identified factors of the extended MMD-HP and extended EDMCQ was measured by Cronbach’s alpha, which ranged from acceptable to very good and from good to very good respectively. The internal consistency was also very good for all items of the original and extended MMD-HP, as well as for all items of the original and extended EDMCQ. More details can be found in Additional file 3.

Descriptive statistics were calculated for demographics and answers to each additional question. Comparisons between intensivists, ICU nurses and supporting staff on continuous variables were made using Analysis of Variance (ANOVA) with Games Howell post hoc tests to correct for multiple testing. The Pearson correlation coefficient was calculated to measure the strength and direction of the association between moral distress levels and several continuous variables expected to be related to moral distress. When testing these Pearson correlations, the Bonferroni method was used to correct for multiple testing. Since seven correlations were examined, overall and for each profession, the associated *p*-values were inflated by multiplying these by 7.

#### Comparison with the historical control group

One year before COVID-19, a nationwide moral distress study using the original MMD-HP was conducted among all ICU-nurses and intensivists working in the Netherlands, of whom 120 intensivists (18.7%) and 499 ICU nurses (10.1%) in the Netherlands completed the questionnaire [21]. In the current study, moral distress outcomes and the highest-ranking moral distress items of the original MMD-HP of intensivists and ICU nurses were compared with this historical control group using independent samples *t* tests.

#### Qualitative analysis

Participants were able to provide narrative commentary by responding to the open-ended questions and comment fields in the questionnaire, which was anonymized down to the subgroup level. Data were analysed using ATLAS.ti v8.4.18 [45]. Qualitative data were analysed independently by two researchers (MD and SP), both medical students. An inductive thematic analysis with the widely accepted six-step process was used to explore deeper themes within the data [46]. First the researchers familiarised themselves with the data (step 1) and generated initial codes (step 2). From these codes common and relevant themes were identified (step 3). Units of comments consisting of one grammatical clause, yet covering different topics were coded as different units. Initial codes were subsumed into more abstract categories, which were eventually collapsed into core-categories using a constant comparative approach (step 4). A codebook was formulated after reaching consensus, which was then used to independently recode the data (step 5). Full coding was initially done by one researcher (MD), while randomly selected parts were double-coded by one other independent researcher (SP). Codes were modified if considered necessary. The pre-final analysis was then checked and any disagreements in coding were resolved by discussion among members of the research team until consensus was reached. Finally, step six of the thematic analysis was completed: reporting the findings. This study was reported according to the Standards for Reporting Qualitative Research [47].

## Results

### Demographic data

The extended MMD-HP was completed by 41 intensivists (5.0%), 355 ICU nurses (9.3%), and 108 supporting staff, the extended EDMCQ by 40 intensivists (4.8%), 345 ICU nurses (9.1%), and 103 supporting staff. Every item was completed for every survey returned. Distribution of gender and age in the intensivist group did not differ significantly from the national population (*p* = 0.71 and *p* = 0.07 respectively). An overview of the participants’ characteristics is given in Table 1.

**Table 1:** Demographics of participants

	Nurse (n = 355)	Intensivist (n = 41)	Supporting staff (n = 108)
Sex			
Male	81 (22.8%)	25 (61.0%)	18 (16.7%)
Female	274 (77.2%)	16 (39.0%)	90 (83.3%)
Mean age	43.64 (11.4)	45.78 (6.7)	40.17 (10.4)
Age categories			
< 30	40 (11.3%)	0 (0.0%)	18 (16.7%)
30–49	177 (73.2%)	30 (73.2%)	65 (60.2%)
≥ 50	138 (38.9%)	11 (26.8%)	25 (23.1%)
Hospital type			
Tertiary, academic	32 (9.0%)	10 (24.4%)	4 (3.7%)
Top referral	189 (53.2%)	20 (48.8%)	77 (71.3%)
Secondary center	131 (36.9%)	11 (26.8%)	27 (25.0%)
Total years of work experience			
< 5	68 (19.2%)	8 (19.5%)	38 (35.8%)
5–19	153 (43.1%)	31 (75.6%)	46 (43.4%)
≥ 20	134 (37.7%)	2 (4.9%)	22 (20.8%)
Years of work experience on current workplace			
< 5	87 (25.5%)	14 (35.9%)	45 (42.9%)
5–19	165 (48.4%)	23 (59.0%)	43 (41.0%)
≥ 20	89 (26.1%)	2 (5.1%)	17 (16.2%)
Normal ICU-bed count			
< 10	47 (13.3%)	7 (17.1%)	7 (6.7%)
10–29	193 (54.7%)	16 (39.0%)	92 (87.6%)
≥ 30	113 (32.0%)	18 (43.9%)	6 (5.7%)
ICU-Bed count during COVID-19			
< 10	15 (4.3%)	0 (0.0%)	4 (3.8%)
10–29	86 (24.5%)	9 (22.0%)	27 (25.5%)
≥ 30	250 (71.2%)	32 (78.0%)	75 (70.8%)
Percentage increase in bed count			
< 20%	48 (13.7%)	3 (7.3%)	19 (18.3%)
20–49%	95 (27.1%)	6 (14.6%)	14 (13.5%)
50–79%	107 (30.5%)	16 (39.0%)	19 (18.3%)
≥ 80%	101 (28.8%)	16 (39.0%)	52 (50.0%)

### Moral distress

#### Overall moral distress level per profession

Moral distress levels were higher for nurses than for supporting staff. Moral distress levels in intensivists did not differ significantly with those of nurses and supporting staff. See Table 2.

**Table 2:** Moral distress score and standard deviation per profession with Games–Howell correction for differences between professions

	Mean (SD)^a^			*p* value		
	Nurse (n = 355)	Intensivist (n = 41)	Supporting staff (n = 108)	N–I^b^	N–S^c^	I–S^d^
Overall moral distress score	2.97 (2.06)	2.56 (1.38)	2.09 (1.71)	0.22	< 0.001	0.19
Factors						
1. Suboptimal patientcare due to organizational restrictions	5.01 (3.45)	3.34 (2.39)	2.77 (2.51)	< 0.001	< 0.001	0.40
2. Inadequate emotional support for patients and their families	5.73 (3.89)	6.20 (3.27)	4.63 (3.91)	0.67	0.030	< 0.05
3. Fear of contamination	3.86 (4.28)	3.59 (3.86)	3.77 (3.86)	0.91	0.98	0.97
4. Collaboration with patients and their families	1.54 (1.75)	1.53 (1.29)	0.78 (1.11)	1.00	< 0.001	< 0.05
5. Culture of fear and hierarchy	0.85 (1.54)	1.03 (1.31)	0.53 (0.91)	0.69	< 0.05	< 0.05
6. Administrative burden	2.96 (3.61)	2.43 (2.63)	2.07 (2.82)	0.47	< 0.05	0.76
7. Disproportional and aimless care	2.68 (2.76)	1.83 (1.55)	1.49 (1.77)	0.010	< 0.001	0.50

^a^Score range 0–16 (higher scores reflect more moral distress)

^b^Nurse vs. intensivist

^c^Nurse vs. supporting staff

^d^Intensivist vs. supporting staff

#### Exploratory factor analysis

EFA of the extended MMD-HP revealed seven factors (see Table 2) which explained 58.8% of the variance.

The factor “Inadequate emotional support for patients and their families” was considered most morally distressing by *all professions* (see Table 2). Of the individual items in this factor, the items “Being unable to provide patients and their families optimal emotional support” and “Being unable to allow patients and their families to have a dignified farewell” received the highest mean scores in *all professions* (see Table 3).

**Table 3:** Highest ranking moral distress situations across nurses for the extended MMD-HP

		Nurses (n = 355)		Intensivists (n = 41)		Supporting staff (n = 108)		
Item no	Item	Mean (SD)^a^	Rank	Mean (SD)	Rank	Mean (SD)	Rank	*p* value
30	Be unable to provide optimal emotional support to anxious and distressed patients/family members	7.69 (5.31)	1	7.22 (4.64)	2	5.38 (5.23)	2	< 0.001
29	Be unable to allow patients/family members to have a dignified farewell	7.00 (5.43)	2	8.02 (4.87)	1	6.55 (5.25)	1	0.32
13	Be required to work with other healthcare team members who are less experienced than patient care requires	6.57 (4.94)	3	3.32 (3.45)	10	2.59 (3.57)	12	< 0.001
16	Be required to care for more patients than I can safely care for	6.22 (4.19)	4	3.05 (3.99)	13	2.42 (4.19)	13	< 0.001
28	Working with other healthcare team members whom I do not know well	6.00 (4.61)	5	2.54 (2.97)	15	4.81 (4.28)	3	< 0.001
17	Experience compromised patient care due to a lack of resources/equipment/bed capacity	5.23 (4.97)	6	6.10 (5.25)	3	3.38 (4.23)	7	0.001
9	Watch patient care suffer because of a lack of provider continuity	5.00 (4.65)	7	3.83 (3.67)	7	3.10 (3.88)	9	< 0.001
35	Providing care to patients of whom the course of the disease and proper treatment is unclear	4.38 (4.24)	8	5.46 (4.76)	4	4.54 (4.94)	4	0.33
32	Feeling obligated to provide care to patients, where the health of my loved ones is at risk	4.30 (5.10)	9	4.22 (4.87)	5	4.37 (4.86)	5	0.99

^a^Score range 0–16 (higher scores reflect more moral distress)

“Fear of contamination” was also experienced as relatively highly morally distressing by *all professions*. The highest scoring item in this factor in *all professionals* was “Feeling obligated to provide care to patients where the health of my loved ones is at risk”.

The mean factor score of “Suboptimal patient care due to organizational restrictions” was particularly high for ICU nurses. “Being required to work with other healthcare team members who are less experienced than patient care requires” and “Being required to care for more patients than I can safely care for” were the highest scoring items in this factor. An overview of items with the highest and lowest scores per factor per profession are shown in Additional file 4.

#### Qualitative comments

Among the 207 qualitative comments regarding the extended MMD-HP, most were related to working with non-standard equipment and resources (16%) and the inability to provide psychosocial care to patients and their families (14%). For example, one ICU nurse commented:

Sometimes I had to beg if the family could visit when the patient was so ill that discontinuing treatment was considered. Rules were regularly eased, but not this rule. (ICU nurse)

ICU nurses in particular frequently reported dilemmas concerning the shortage of nursing staff. Among the 106 comments from ICU nurses, 18 related to a suboptimal level of competence of colleagues and 14 to the high number of patients they had to care for. Other frequently reported comments from ICU nurses concerned poor communication about rules, regulations and guidelines by their supervisors (n = 18). One nurse wrote:

Regulations are sometimes changed per half-day. It is impossible to keep up. (ICU nurse)

#### Associations between moral distress, ethical climate and respondents’ characteristics

Response-percentages of each professional group per additional question are provided in Additional file 5. Higher moral distress scores were associated with lower ethical climate scores in ICU nurses (r =  − 0.55, *p* < 0.001) and supporting staff (r =  − 0.47, *p* < 0.001). No significant correlation was found for intensivists. As shown in Table 4, ICU professionals who considered quitting their position reported significantly higher moral distress scores than those who did not (*p* < 0.001). Higher moral distress scores were likewise seen in participants considering using psychosocial help (*p* < 0.001) and in participants who indicated that the attention paid to moral distress was insufficient (*p* < 0.001). A greater number of ICU beds was significantly correlated with higher moral distress scores in ICU nurses (r = 0.21, *p* < 0.001), but not for intensivists or supporting staff. No statistically significant correlation was found for moral distress regarding age and work experience for all professions.

**Table 4:** Associations between moral distress and respondents’ characteristics

Variable	n (%)	Moral distress score (SD)
Not considering leaving job now^a^	470 (93.1)	2.57 (1.80)
Considering leaving job now	35 (6.9)	5.03 (2.63)
Enough attention is paid to moral distress^b^	410 (81.7)	2.44 (1.72)
Not enough attention is paid to moral distress	92 (18.3)	4.10 (2.37)
Not considering using psychosocial help now^c^	450 (93.8)	2.65 (1.92)
Considering using psychosocial help now	30 (6.3)	4.05 (2.25)

^a^Not considering leaving now vs. considering leaving now (p < 0.001)

^b^Enough attention is paid vs. not enough attention is paid (p < 0.001)

^c^Not considering using help now vs. considering using help now (p < 0.001)

#### Services addressing moral distress

Most participants (93.4%) declared that services providing support for moral distress were implemented during COVID-19. A total of 764 narrative comments were received to the question what services were provided to address moral distress. The provision of professional moral distress support was most frequently reported (60%), e.g., support by psychologists, social workers, spiritual counsellors, peer supporters, and other mental health support team members. Furthermore, an “open” culture in which managers are approachable and professional problems and feelings or emotions could be shared with colleagues was frequently mentioned (18%). Debriefing as an intervention was also reported by all professions (15%), sometimes in the presence of a psychosocial support worker.

#### Suggestions regarding the reduction of moral distress

Of all respondents, 18.4% indicated that the attention paid to addressing moral distress was insufficient. Respondents provided 154 suggestions how to address moral distress. Some suggested to make psychosocial support more accessible and individually tailored and to continue offering this support after the crisis is over, as one supporting staff member describes:

Aftercare will be necessary. We now find ourselves in the eye of the storm. When the worst is over, we will realize what happened.

Others proposed to improve mutual respect and support within the team by showing more appreciation by the supervisor and sharing experiences and feelings. Furthermore, respondents suggested to organize more frequent evaluations and to improve interdisciplinary shared decision making, for example by increasing involvement of nurses in decision making.

Too little attention was paid to relevant questions from the nursing ward. There was a clear top-down atmosphere, probably due to time constraints. I felt my voice was not being heard, even though my usual focus is infection prevention. (ICU nurse)

Some described how working conditions should be improved. Some ICU nurses for example called upon financial investments from the government to improve working conditions. Respondents furthermore described how they wanted to be able to provide better psychosocial care to patients and their families.

There should be more room for families to visit patients, by providing more and better protective measures. Better contact and more dignified patient care would reduce much moral distress. (ICU nurse)

### Ethical climate

#### Exploratory factor analysis

EFA of the extended EDMCQ revealed eight factors, which explained 61.8% of the variance.

#### Ethical climate per profession

While the ethical climate factor scores of ICU nurses and supporting staff were mostly comparable, intensivists had significantly higher ethical climate scores on all factors except for “Practice and culture of ethical awareness and support”. “Practice and culture of ethical awareness and support” and “Culture of mutual respect within the interdisciplinary team” were the highest perceived ethical climate factors in *all professions*. “Culture of not avoiding end-of-life-decisions” and “Self-reflective and empowering leadership” received the lowest mean scores (see Table 5). “Patients with little chance of recovery are not frequently admitted” and “Physicians in charge dare to show their vulnerability” were the lowest scoring ethical climate items in *all professions*. Additional file 6 shows the lowest ranking ethical climate items in the extended EMDCQ per profession.

**Table 5:** Ethical climate score per profession per factor with Games–Howell correction for differences between professions

	Mean (SD)^a^			P-value		
	Nurse (n = 345)	Intensivist (n = 40)	Supporting staff (n = 103)	N–I^b^	N–S^c^	I–S^d^
Overall ethical climate score	3.90 (0.54)	4.11 (0.38)	3.89 (0.43)	< 0.05	0.96	< 0.05
Factor						
1. Practice and culture of ethical awareness and support	4.34 (0.59)	4.23 (0.60)	4.23 (0.62)	0.56	0.30	1.00
2. Self-reflective and empowering leadership by physicians	3.48 (0.67)	3.74 (0.51)	3.52 (0.54)	< 0.05	0.76	< 0.05
3. Culture of not avoiding end-of-life decisions	3.36 (0.91)	3.81 (0.75)	3.40 (0.65)	< 0.05	0.97	< 0.05
4. Practice and culture of open interdisciplinary reflection/discussion	3.74 (0.85)	4.14 (0.61)	3.85 (0.70)	< 0.05	0.40	< 0.05
5. Active involvement of nurses in end-of-life care and decision making	3.73 (0.65)	4.02 (0.56)	3.77 (0.58)	< 0.05	0.83	< 0.05
6. Relaxation after or during work	3.80 (0.84)	4.12 (0.60)	4.00 (0.67)	< 0.05	< 0.05	0.52
7. Culture of mutual respect within the interdisciplinary team	4.34 (0.72)	4.63 (0.52)	4.04 (0.81)	< 0.05	< 0.05	< 0.001
8. Active decision making by physicians	4.22 (0.77)	4.58 (0.59)	4.19 (0.67)	< 0.05	0.94	< 0.05

^a^Score range 1–5 (higher scores reflect better perceived ethical climate)

^b^Nurse vs. intensivist

^c^Nurse vs. supporting staff

^d^Intensivist vs. supporting staff

#### Qualitative comments

From the 504 respondents 157 narrative comments to the open ended questions relating to the ethical climate were received, respectively by 133 ICU nurses, 8 intensivists and 36 supporting staff. Comments concerning team climate (n = 42) were mainly related to the themes of a lack of interdisciplinary shared decision making (48%), as illustrated below:

Nurses no longer participate in the multidisciplinary consultation. No ifs or buts, just work! (ICU nurse)

Also, a lack of reflective practice (21%), and feeling undervalued (17%) were often mentioned in the context of the team climate.

While 18% of the comments on leadership culture (n = 37) indicated satisfaction, some described hierarchy (22%).

We are not equals… certainly not in a crisis situation. (ICU nurse)

Others found there was a lack of self-reflective and firm leadership (10%).

The problems regarding psychosocial health (n = 46) that participants most frequently reported were fatigue (56%) and sleeping difficulty (36%), as one described:

Especially in the onset of the crisis, I had many sleepiness nights as I couldn’t stop worrying. I had thoughts like: when will it start? How bad will it get? How long will it take? Will I get sick? Will my family get sick? Will we end up in the same situation as Italy? (ICU nurse)

Other comments were related to hectic in the ICU during the peak of the pandemic (33%). Comments on EOL-care (n = 15) were related to the unpredictability of the COVID-19 disease (25%), postponement of EOL-decisions (8%), and not being involved in decision making (8%).

End-of-life care during COVID-19 is very different from normal… there is no consultation between physicians and nurses. (ICU nurse)

### Comparison with the historical control group

When comparing the *original* MMD-HP with the historical control group, the total mean moral distress score for ICU nurses (2.35 versus 3.00, *p* < 0.001) and intensivists (1.91 versus 2.67, *p* < 0.05) decreased significantly.

A comparison of the *original* MMD-HP was made for the most morally distressing items per profession during COVID-19 and the historical control group one year before COVID-19 (see Additional file 7). For nurses this comparison revealed significant differences, while in intensivists the stress score is comparable to one year prior. In particular, “Be required to work with colleagues who are less experienced than patient care requires” and “Caring for more patients than I can safely care for” received significant higher stress scores in ICU nurses than one year prior.

## Discussion

This study is the first nationwide survey regarding moral distress among intensive care professionals in the Netherlands during the COVID-19 pandemic and also the first comparison with a national historical control group one year before the COVID-19 outbreak.

The main results are discussed below.

### Moral distress

While in pre-COVID-19 studies nurses had higher moral distress levels than intensivists, our results show that these differences have decreased during the pandemic [7, 21]. However, nurses had significantly higher moral distress levels than supporting staff. The moral distress scores reported are relatively small. However, some situations did not or rarely occur as reported by the participants, and therefore the impact of these situations regarding moral distress is minimal or absent.

Different root causes for moral distress among ICU professionals have been identified. Our findings underscore that the inability to provide psychosocial care to patients and their families was a major problematic issue for all professions. Families were banned from visiting their seriously ill and dying loved ones due to the need for social distancing. This may not only feel inhuman, but has also adverse consequences for shared decision making about treatment and EOL-care [48, 49]. As previously described [35], during the COVID pandemic, a constantly impending dilemma of safe working conditions versus high quality of care occurred. ICU professionals were afraid of becoming infected and worried about the consequences of the latter for themselves and their environment. Furthermore, our respondents frequently reported they experienced moral distress from working in unfamiliar working environments and participating in unfamiliar processes, which is known to have consequences for quality and safety of patient care [50, 51]. In addition, the communication by supervisors was reported to be poor, which is in line with other studies [29, 32]. However, transparent communication in a crisis situation is known to be challenging as new policies have to be constantly adapted and implemented.

Both the ranking of morally distressing situations and the response to the open-ended questions show that ICU nurses often experienced moral distress from caring for too many patients. ICU nurses had to care for three (85%) or four (36%) patients during the first peak of the pandemic instead of applying the usual one nurse to one patient ratio [52]. The contemporary and increasing shortage of nursing staff worldwide has become even more pronounced and problematic during the pandemic [53]. To address the shortage of ICU staff and increase the ICU bed capacity during the pandemic, personnel from other departments, who were not primarily qualified to deliver ICU care, were temporarily deployed. However, this study confirms that working with less trained team members is particularly morally distressing for ICU nurses [35]. This may increase the symptoms of burnout [35], which puts the ICU care in an untenable situation.

Respondents provided multiple suggestions to address moral distress. Supportive engagement, a clear vision and good feedback to healthcare professionals from supervisors is needed to protect against psychological outcomes [54]. Regular and structural debriefing sessions have been proven to mitigate moral distress in ICU nursing staff [55] and is expected to improve shared decision making. Furthermore, respondents voiced their need for better accessible and individually tailored psychosocial support. However, in this study, 18.4% of all respondents reported that the attention paid to moral distress was insufficient, compared to 67.7% in 2019. ICU management and the government seem to have made progress in recognizing and addressing the hazards of moral distress within healthcare settings. As higher moral distress scores were seen in ICU professionals who declared to consider using professional psychosocial help, it is interesting to discuss what the particular needs different groups of professionals have and by what group of professionals psychosocial support should be provided.

### Ethical climate

This study corroborates the negative association between moral distress levels and healthier ethical climates in nurses and supporting staff [7], but not in intensivists. Perhaps this is due to the number of intensivists that participated. Our findings suggest that a feeling of solidarity prevailed during COVID-19. Nevertheless, leadership should be more self-reflective and empowering. A remarkable issue is the large number of ICU professionals who reported being fatigued and having sleeping difficulties. A recent review on the prevalence of depression, anxiety and insomnia among healthcare workers, reported that one in three healthcare workers suffered from insomnia during COVID-19 [56]. These results underscore the relevance of this recently recognized problem associated with a significant risk of morbidity [57].

### Comparison with the historical control group

Strikingly, the overall moral distress score for ICU nurses and intensivists during the COVID-19 pandemic turned out to be significantly *lower* compared to the historical control group. We postulate several possible explanations. First, moral distress levels might have been higher very early during the pandemic, when there was much uncertainty about the course of the disease and little organizational control. Second, ICU professionals may have gone into survival mode as a strategy to make it through the crisis, neglecting their own mental health needs. In this perspective, moral distress levels might have increased sometime after this stressful period. Third, proactively provided professional psychosocial care and the feeling of solidarity that prevailed during the crisis may have reduced the feeling of moral distress. Furthermore, selection bias might have occurred in that only the least morally distressed professionals participated. Lastly, it can be hypothesized that factors that elicit high moral distress levels during normal circumstances may apply less during a crisis. Since the additional factors emerging from the extra items received relatively high moral distress scores in all professions, the *extended* MMD-HP may be more valid to measure moral distress during a pandemic than the original MMD-HP. The revised version is expert and psychometrically validated and can perhaps be used in future studies.

### Limitations

Limitations of this study include a limited response rate, which could have resulted in selection bias. Especially the limited number of participating intensivists hampers the interpretation of responses. However, there were no indications of non-response bias for this group. As data on the age and gender distribution of ICU nurses and supporting staff were unavailable, it is unclear whether the respondents of these professions are representative for their entire population. Furthermore, interregional differences of moral distress levels were not taken into account, although the pressure on critically ill patient care delivery and experienced moral distress is likely to have differed between Dutch regions [58]. In addition, moral distress among ICU professionals in the Netherlands during COVID-19 may differ from that in other countries. Replicating the study in other countries would be necessary to establish its generalizability.

## Conclusions

ICU professionals in the Netherlands have experienced moral distress to varying degrees during the COVID-19 pandemic. All professional groups experienced high levels of moral distress from the inability of providing psychosocial support for patients and their families, but other causes of moral distress differed. The overall moral distress scores of the original MMD-HP were significantly *lower* in ICU nurses and intensivists compared to the historical control group one year before COVID-19. However, it is questionable whether the original MMD-HP is an adequate tool to measure moral distress during a pandemic. The revised version is sufficiently reliable and valid and can be used in future studies. To address moral distress during a crisis, leaders must provide a supportive environment, communicate clearly, and encourage group reflective debriefings. By minimizing moral distress, intensive care professionals’ mental health and staff retention, as well as patient care quality and safety, increase.

## Supplementary Information

**Additional file 1**. Questionnaire copy. Copy of full questionnaire that is used.

**Additional file 2**. Identified factors and factor loadings. Tables with identified factors and factor loadings per item of the extended MMD-HP and EDMCQ.

**Additional file 3**. Exploratory factor analysis and internal consistency. Tables with identified factors, number of items, percentage of variance, mean scale score and Cronbach’s alpha explained by each factor of the extended MMD-HP and EDMCQ.

**Additional file 4**. Highest and lowest scoring items per factor. Overview of items with the highest and lowest scores per factor of the extended MMD-HP for ICU nurses, intensivists and supporting staff.

**Additional file 5**. Response-percentages additional questions. Response-percentages of each professional group per additional question.

**Additional file 6**. Lowest ranking ethical climate items. Overview of the lowest ranking ethical climate items in the extended EDMCQ per profession.

**Additional file 7**. Most morally distressing items compared with 2019. Comparison of the original MMD-HP for the most morally distressing items per profession during COVID-19 and the historical control group one year before COVID-19.

## Data Availability

The datasets used and/or analysed during the current study are available from the corresponding author on reasonable request.

## Abbreviations

ICU: Intensive care unit
MMD-HP: Measurement of moral distress for healthcare professionals
EDMCQ: Ethical decision-making climate questionnaire
EOL: End-of-life
EFA: Exploratory factor analysis
ANOVA: Analysis of variance

## References

[1] Gostin LO. Pandemic influenza: public health preparedness for the next global health emergency. J Law Med Ethics 2004;32(4):565–73. 10.1111/j.1748-720x.2004.tb01962.x. 10.1111/j.1748-720x.2004.tb01962.x 15807345

[2] Holt GR (2008). Making difficult ethical decisions in patient care during natural disasters and other mass casualty events. Otolaryngol Head Neck Surg.

[3] Wynia MK (2007). Ethics and public health emergencies: encouraging responsibility. Am J Bioethics.

[4] Mastroianni AC, Kahn JP, Kass NE. An overview of public health ethics in emergency preparedness and response. In: The Oxford handbook of public health ethics. Oxford University Press; 2019. Retrieved Apr 28 2020, from https://www.oxfordhandbooks.com/view/10.1093/oxfordhb/9780190245191.001.0001/oxfordhb-9780190245191-e-66.

[5] Gammon J, Hunt J (2018). Source isolation and patient wellbeing in healthcare settings. Br J Nurs.

[6] Jameton A (1984). Nursing practice: the ethical issues.

[7] Whitehead PB, Herbertson RK, Hamric AB, Epstein EG, Fisher JM (2015). Moral distress among healthcare professionals: report of an institution-wide survey. J Nurs Scholarsh.

[8] Pauly B, Varcoe C, Storch J, Newton L (2009). Registered nurses' perceptions of moral distress and ethical climate. Nurs Ethics.

[9] Altaker KW, Howie-Esquivel J, Cataldo JK (2018). Relationships among palliative care, ethical climate, empowerment, and moral distress in intensive care unit nurses. Am J Crit Care.

[10] Lamiani G, Borghi L, Argentero P (2017). When healthcare professionals cannot do the right thing: a systematic review of moral distress and its correlates. J Health Psychol.

[11] Karanikola MN, Albarran JW, Drigo E, Giannakopoulou M, Kalafati M, Mpouzika M (2014). Moral distress, autonomy and nurse-physician collaboration among intensive care unit nurses in Italy. J Nurs Manag.

[12] Dalmolin Gde L, Lunardi VL, Lunardi GL, Barlem EL, Silveira RS. Moral distress and burnout syndrome: Are there relationships between these phenomena in nursing workers? Rev Lat Am Enfermagem 2014;22(1):35–42. 10.1590/0104-1169.3102.2393. 10.1590/0104-1169.3102.2393 PMC4292705 24553701

[13] Hamaideh SH (2014). Moral distress and its correlates among mental health nurses in Jordan. Int J Ment Health Nurs.

[14] Ganz FD, Raanan O, Khalaila R, Bennaroch K, Scherman S, Bruttin M (2013). Moral distress and structural empowerment among a national sample of Israeli intensive care nurses. J Adv Nurs.

[15] Browning AM (2013). CNE article: moral distress and psychological empowerment in critical care nurses caring for adults at end of life. Am J Crit Care.

[16] Wiegand DL, Funk M (2012). Consequences of clinical situations that cause critical care nurses to experience moral distress. Nurs Ethics.

[17] Özden D, Karagözoğlu Ş, Yildirim G (2013). Intensive care nurses' perception of futility: job satisfaction and burnout dimensions. Nurs Ethics.

[18] Pavlish C, Brown-Saltzman K, Hersh M, Shirk M, Rounkle AM (2011). Nursing priorities, actions, and regrets for ethical situations in clinical practice. J Nurs Scholarsh.

[19] Varcoe C, Pauly B, Storch J, Newton L, Makaroff K (2012). Nurses' perceptions of and responses to morally distressing situations. Nurs Ethics.

[20] Van den Bulcke B, Metaxa V, Reyners AK, Rusinova K, Jensen HI, Malmgren J, et al.; DISPROPRICUS study group of the Ethics Section of the ESICM. Ethical climate and intention to leave among critical care clinicians: an observational study in 68 intensive care units across Europe and the United States. Intensive Care Med. 2020;46(1):46–56. 10.1007/s00134-019-05829-1. 10.1007/s00134-019-05829-1 PMC6954133 31690968

[21] Gilissen V, Donkers M, Van Dijk N, Kling H, Van Santen S, Klaassens M, et al. Moral distress and ethical climate in intensive care medicine: a nationwide study among Dutch ICU-nurses, -specialists and -fellows. [Submitted].

[22] Wu P, Fang Y, Guan Z, Fan B, Kong J, Yao Z (2009). The psychological impact of the SARS epidemic on hospital employees in China: exposure, risk perception, and altruistic acceptance of risk. Can J Psychiatry.

[23] Chong MY, Wang WC, Hsieh WC, Lee CY, Chiu NM, Yeh WC (2004). Psychological impact of severe acute respiratory syndrome on health workers in a tertiary hospital. Br J Psychiatry.

[24] Liu X, Kakade M, Fuller CJ, Fan B, Fang Y, Kong J (2012). Depression after exposure to stressful events: lessons learned from the severe acute respiratory syndrome epidemic. Compr Psychiatry.

[25] Lancee WJ, Maunder RG, Goldbloom DS; Coauthors for the Impact of SARS Study. Prevalence of psychiatric disorders among Toronto hospital workers one to two years after the SARS outbreak. Psychiatr Serv 2008;59(1):91–5. 10.1176/ps.2008.59.1.91. 10.1176/ps.2008.59.1.91 PMC2923654 18182545

[26] Styra R, Hawryluck L, Robinson S, Kasapinovic S, Fones C, Gold WL (2008). Impact on health care workers employed in high-risk areas during the Toronto SARS outbreak. J Psychosom Res.

[27] Maunder RG, Lancee WJ, Balderson KE, Bennett JP, Borgundvaag B, Evans S (2006). Long-term psychological and occupational effects of providing hospital healthcare during SARS outbreak. Emerg Infect Dis.

[28] Maunder R (2004). The experience of the 2003 SARS outbreak as a traumatic stress among frontline healthcare workers in Toronto: lessons learned. Philos Trans R Soc Lond B Biol Sci.

[29] Wahlster S, Sharma M, Lewis AK, Patel PV, Hartog CS, Jannotta G (2021). The coronavirus disease 2019 pandemic's effect on critical care resources and health-care providers: a global survey. Chest.

[30] Lasalvia A, Amaddeo F, Porru S, Carta A, Tardivo S, Bovo C (2021). Levels of burn-out among healthcare workers during the COVID-19 pandemic and their associated factors: a cross-sectional study in a tertiary hospital of a highly burdened area of north-east Italy. BMJ Open.

[31] Chen R, Sun C, Chen JJ, Jen HJ, Kang XL, Kao CC (2021). A large-scale survey on trauma, burnout, and posttraumatic growth among nurses during the COVID-19 pandemic. Int J Ment Health Nurs.

[32] González-Gil MT, González-Blázquez C, Parro-Moreno AI, Pedraz-Marcos A, Palmar-Santos A, Otero-García L (2021). Nurses' perceptions and demands regarding COVID-19 care delivery in critical care units and hospital emergency services. Intensive Crit Care Nurs.

[33] Caillet A, Coste C, Sanchez R, Allaouchiche B (2020). Psychological Impact of COVID-19 on ICU Caregivers. Anaesth Crit Care Pain Med.

[34] Azoulay E, Cariou A, Bruneel F, Demoule A, Kouatchet A, Reuter D, et al. Symptoms of anxiety, depression, and peritraumatic dissociation in critical care clinicians managing patients with COVID-19. A cross-sectional study. Am J Respir Crit Care Med. 2020;202(10):1388–1398. 10.1164/rccm.202006-2568OC. 10.1164/rccm.202006-2568OC PMC7667906 32866409

[35] Kok N, van Gurp J, Teerenstra S, van der Hoeven H, Fuchs M, Hoedemaekers C (2021). Coronavirus disease 2019 immediately increases burnout symptoms in ICU professionals: a longitudinal cohort study. Crit Care Med.

[36] Nationale Intensive Care Evaluatie (NICE). COVID-19 infecties op de IC’s. https://www.stichting-nice.nl/. Accessed 14 March 2021.

[37] Qualtrics. (Version April, 2020). Provo, Utah, USA: Qualtrics. (2005). Available at: http://www.qualtrics.com.

[38] Epstein EG, Whitehead PB, Prompahakul C, Thacker LR, Hamric AB. Enhancing understanding of moral distress: the measure of moral distress for health care professionals. AJOB Empir Bioeth 2019;10(2):113–124. 10.1080/23294515.2019.1586008. 10.1080/23294515.2019.1586008 31002584

[39] Van den Bulcke B, Piers R, Jensen HI, Malmgren J, Metaxa V, Reyners AK (2018). Ethical decision-making climate in the ICU: theoretical framework and validation of a self-assessment tool. BMJ Qual Saf.

[40] Cao J, Wei J, Zhu H, Duan Y, Geng W, Hong X (2020). A study of basic needs and psychological wellbeing of medical workers in the fever clinic of a tertiary general hospital in Beijing during the COVID-19 outbreak. Psychother Psychosom.

[41] Chung JPY, Yeung WS (2020). Staff mental health self-assessment during the COVID-19 outbreak. East Asian Arch Psychiatry.

[42] Huang JZ, Han MF, Luo TD, Ren AK, Zhou XP. [Mental health survey of medical staff in a tertiary infectious disease hospital for COVID-19]. Zhonghua Lao Dong Wei Sheng Zhi Ye Bing Za Zhi 2020;38(3):192–195. 10.3760/cma.j.cn121094-20200219-00063. 10.3760/cma.j.cn121094-20200219-00063 32131151

[43] Lai J, Ma S, Wang Y, Cai Z, Hu J, Wei N, et al. Factors associated with mental health outcomes among health care workers exposed to coronavirus disease 2019. JAMA Netw Open. 2020;3(3):e203976. 10.1001/jamanetworkopen.2020.3976. 10.1001/jamanetworkopen.2020.3976 PMC7090843 32202646

[44] IBM SPSS Statistics for Windows. 25.0.0.1 ed. Armonk, NY: IBM Corp; 2017.

[45] Atlas.ti. 8.4.18 ed. Berlin: Atlas.ti Scientific Software Development GmbH; 2019.

[46] Kiger ME, Varpio L. Thematic analysis of qualitative data: AMEE Guide No. 131. Med Teach 2020;42(8):846–854. 10.1080/0142159X.2020.1755030. 10.1080/0142159X.2020.1755030 32356468

[47] O'Brien BC, Harris IB, Beckman TJ, Reed DA, Cook DA (2014). Standards for reporting qualitative research: a synthesis of recommendations. Acad Med.

[48] Simpson N, Milnes S, Steinfort D (2020). Don’t forget shared decision-making in the COVID-19 crisis. Intern Med J.

[49] Rajagopal MR. To comfort always: are we ignoring this duty in covid protocols? Indian J Med Ethics 2020;5:189–91. 10.20529/IJME.2020.071. 10.20529/IJME.2020.071 33295287

[50] Aiken LH, Sloane DM, Bruyneel L, Van den Heede K, Sermeus W; RN4CAST Consortium. Nurses' reports of working conditions and hospital quality of care in 12 countries in Europe. Int J Nurs Stud. 2013;50(2):143–53. 10.1016/j.ijnurstu.2012.11.009. 10.1016/j.ijnurstu.2012.11.009 23254247

[51] Moisoglou I, Yfantis A, Galanis P, Pispirigou A, Chatzimargaritis E, Theoxari A (2020). Nurses work environment and patients' quality of care. Int J Caring Sci.

[52] Beroepsvereniging Verzorgenden Verpleegkundigen (V&VN). Peiling IC-verpleegkundigen: snel naar 1.700 IC-bedden structureel niet realistisch. 2020, May 19. Retrieved 26 June 2020, from https://www.venvn.nl/nieuws/peiling-ic-verpleegkundigen-snel-naar-1-700-ic-bedden-structureel-niet-realistisch/.

[53] World Health Organization. State of the World’s Nursing: Investing in Education, Jobs and Leadership. WHO: Geneva; 2020. Retrieved 28 sept 2020, from https://www.who.int/publications-detail/nursing-report-2020.

[54] Brooks SK, Dunn R, Sage CA, Amlôt R, Greenberg N, Rubin GJ (2015). Risk and resilience factors affecting the psychological wellbeing of individuals deployed in humanitarian relief roles after a disaster. J Ment Health.

[55] Browning ED, Cruz JS. Reflective debriefing: a social work intervention addressing moral distress among ICU nurses. J Soc Work End Life Palliat Care 2018;14(1):44–72. doi: 10.1080/15524256.2018.1437588. 10.1080/15524256.2018.1437588 29488856

[56] Pappa S, Ntella V, Giannakas T, Giannakoulis VG, Papoutsi E, Katsaounou P (2020). Prevalence of depression, anxiety, and insomnia among healthcare workers during the COVID-19 pandemic: a systematic review and meta-analysis. Brain Behav Immun.

[57] Sivertsen B, Lallukka T, Salo P, Pallesen S, Hysing M, Krokstad S, Simon Øverland. Insomnia as a risk factor for ill health: results from the large population-based prospective HUNT Study in Norway. J Sleep Res 2014;23(2):124–32. 10.1111/jsr.12102. 10.1111/jsr.12102 24635564

[58] National Institute for Public Health and the Environment. Ministry of Health, Welfare and Sport. Regional differences in the coronavirus epidemic. RIVM: Bilthoven, Apr 4 2020. Retrieved June 25 2020, from https://www.rivm.nl/en/news/regional-differences-in-coronavirus-epidemic.

