# Supplementary material for: Moral distress and ethical climate in intensive care medicine during COVID-19: a nationwide study
Source: BMC Med Ethics. 2021 Jun 17;22:73. doi: 10.1186/s12910-021-00641-3 (PMC8211309; doi:10.1186/s12910-021-00641-3)
Supplement: Supplementary file 1 — Additional file 1. Questionnaire copy. Copy of full questionnaire that is used. [file 12910_2021_641_MOESM1_ESM.docx]

**ADDITIONAL FILE 1: questionnaire copy**

**Extended Moral Decision Making – Healthcare Professionals (MMD-HP) Questionnaire**

Moral distress occurs when professionals cannot carry out what they believe to be ethically appropriate actions because of constraints or barriers. This survey lists situations that occur in clinical practice. If you have experienced these situations, they may or may not have been morally distressing to you. Please indicate how frequently you have experienced each item. Also, rank how distressing these situations are for you. If you have never experienced a particular situation, select "0" (never) for frequency. Even if you have not experienced a situation, please indicate how distressed you would be if it occurred in your practice. Note that you will respond to each item by checking the appropriate column for two dimensions: Frequency and Level of Distress.

| Items | Frequency | Level of Distress |
| --- | --- | --- |
|  | Never (0) - Very frequently (4) | None (0) Very distressing (4) |
| 1. Witness healthcare providers giving “false hope” to a patient or family. |  |  |
| 1. Follow the family’s insistence to continue aggressive treatment even though I believe it is not in the best interest of the patient. |  |  |
| 1. Feel pressured to order or carry out orders for what I consider to be unnecessary or inappropriate tests and treatments. |  |  |
| 1. Be unable to provide optimal care due to pressures from administrators or insurers to reduce costs. |  |  |
| 1. Continue to provide aggressive treatment for a person who is most likely to die regardless of this treatment when no one will make a decision to withdraw it. |  |  |
| 1. Be pressured to avoid taking action when I learn that a physician, nurse, or other team colleague has made a medical error and does not report it. |  |  |
| 1. Be required to care for patients whom I do not feel qualified to care for. |  |  |
| 1. Participate in care that causes unnecessary suffering or does not adequately relieve pain or symptoms. |  |  |
| 1. Watch patient care suffer because of a lack of provider continuity. |  |  |
| 1. Follow a physician’s or family member’s request not to discuss the patient’s prognosis with the patient/family. |  |  |
| 1. Witness a violation of a standard of practice or a code of ethics and not feel sufficiently supported to report the violation. |  |  |
| 1. Participate in care that I do not agree with but do so because of fears of litigation. |  |  |
| 1. Be required to work with other healthcare team members who are less experienced than patient care requires. |  |  |
| 1. Witness low quality of patient care due to poor team communication. |  |  |
| 1. Feel pressured to ignore situations in which patients have not been given adequate information to ensure informed consent. |  |  |
| 1. Be required to care for more patients than I can safely care for. |  |  |
| 1. Experience compromised patient care due to lack of resources/equipment/bed capacity. |  |  |
| 1. Experience lack of administrative action or support for a problem that is compromising patient care. |  |  |
| 1. Have excessive documentation requirements that compromise patient care. |  |  |
| 1. Fear retribution if I speak up. |  |  |
| 1. Feel unsafe/bullied amongst my own colleagues. |  |  |
| 1. Be required to work with abusive patients/family members who are compromising quality of care. |  |  |
| 1. Feel required to overemphasize tasks and productivity or quality measures at the expense of patient care. |  |  |
| 1. Be required to care for patients who have unclear or inconsistent treatment plans or who lack goals of care. |  |  |
| 1. Work within power hierarchies in teams, units, and my institution that compromise patient care. |  |  |
| 1. Participate on a team that gives inconsistent messages to a patient/family. |  |  |
| 1. Work with team members who do not treat vulnerable or stigmatized patients with dignity and respect. |  |  |
| 1. Working with other healthcare team members whom I do not know well. |  |  |
| 1. Be unable to allow patients/family members to have a dignified farewell. |  |  |
| 1. Be unable to provide optimal emotional support to anxious and distressed patients/family members. |  |  |
| 1. Feeling obligated to provide care to patients where my own health is at risk. |  |  |
| 1. Feeling obligated to provide care to patients where the health of my loved ones is at risk. |  |  |
| 1. Feeling unsafe due to a limited stock of protective equipment, such as mouth masks, gowns, safety glasses, aprons, gloves and/or disinfectants. |  |  |
| 1. Be unable to provide family/patient with consistent information, for example due to lack of time or communication facilities. |  |  |
| 1. Providing care to patients of whom the course of the disease and proper treatment is unclear. |  |  |
| If there are other situations in which you have felt moral distress, please write and score them here: |  |  |
| 1. |  |  |
| 2. |  |  |

Have you ever left or considered leaving a clinical position due to moral distress?

- - No, I have never considered leaving or left a position.
  - Yes, I considered leaving but did not leave.
  - Yes, I left a position.

Are you considering leaving your position now due to moral distress?

- Yes
- No

Is there paid attention to moral distress in your hospital during the COVID-19 crisis?

- Yes
- No
- I don’t know

If yes: in what way has moral distress been addressed?

In your opinion, has moral distress been addressed sufficiently in your hospital during the COVID-19 crisis?

- Yes
- No

How do you think moral distress could be better addressed?

**Extended Ethical Decision-Making Climate Questionnaire (EDMCQ)**

The following questions are about the ethical climate in your department at the time of the COVID-19 crisis.

The statements below are about the *team climate* in your department at the time of the COVID-19 crisis. Indicate to what extent you agree with the statement.

In my ICU….

1. … There are regular opportunities for open informal dialogue between healthcare providers.
2. … There is regular structured and formal dialogue between the various disciplines within the team to discuss patient care.
3. … We regularly reflect on the quality of care provided from the various points of view of the staff.
4. … The teams are well coordinated/managed.
5. … There is an open and constructive culture in the department such that criticism can be easily expressed.
6. … Discussions about patients lead to greater understanding and agreements.
7. … I am always regarded and addressed by everyone in the team as a full-fledged team member.
8. … Team members from another discipline respect my work.
9. … I have confidence in the professional competence of my team members.
10. ... the culture in the department makes it easy to learn from the mistakes of others.

Any comments on the above statements:

Below are some questions about the *leadership culture* in your department.

Indicate how often the situation occurs at the time of the COVID-19 crisis.

In my ICU…

1. … Physicians in charge make accurate and timely decisions
2. … Physicians in charge take full charge when emergencies arise.
3. … Physicians in charge are not hesitant about taking initiative in the group.
4. … Physicians in charge help team members settle their differences.
5. … Physicians in charge trust the team members to exercise good judgement.
6. … Physicians in charge permit the team members to use their own judgement in solving problems.
7. … Physicians in charge encourage initiative in the team members.
8. … Physicians in charge treat all team members as their equals
9. … Physicians in charge are well aware of their own emotions and attitudes
10. … Physicians in charge are well aware of their role model function.
11. … Physicians in charge dare to show their vulnerability

Any comments on the above statements:

Below are some questions about *end-of-life care* at the time of the COVID-19 crisis.

Indicate to what extent you agree with the statement.

In my ICU…

1. ... my colleagues understand my ideas / feelings about difficult end-of-life decisions.
2. ... different opinions and values are tolerated.
3. ... we talk about moral and ethical problems.
4. … There is a structured, formal debriefing after difficult patient care situation
5. … Nurses are present during the communication of end-of-life information to the family.
6. … Nurses are involved in end-of-life decision-making.
7. … Nurses and physicians collaborate well with one another during end-of-life situations.
8. … Death is perceived as a treatment failure, so decisions to withdraw or withhold therapy are rarely made.
9. … EOL decisions are frequently postponed.
10. … Patients with little chance of recovery are frequently admitted.
11. … Patients with little chance of recovery do frequently occupy an ICU bed which other patients would benefit more from

Any comments on the above statements:

The statements below are about the *psychosocial health* in your department at the time of the COVID-19 crisis. Indicate to what extent you agree with the statement.

In my ICU…

1. … I can vent my heart
2. … experiences and concerns are shared with each other
3. … sufficient attention is paid to the impact of the situation on each other
4. … Colleagues regularly inquire about my concerns and needs.
5. … I know where I can go for professional psychosocial support.
6. … Differences in personal or family circumstances of colleagues are respected
7. … Differences in culture and religion of colleagues are respected
8. .… I have enough breaks to eat, drink and relax.
9. .… I have enough time for relaxation next to work.
10. … there is calmness, control and overview in the department.
11. .… I can adequately focus on my work during my shifts.
12. .… I have enough sleep to be rested at work.
