# Supplementary material for: Moral distress and ethical climate in intensive care medicine during COVID-19: a nationwide study
Source: BMC Med Ethics. 2021 Jun 17;22:73. doi: 10.1186/s12910-021-00641-3 (PMC8211309; doi:10.1186/s12910-021-00641-3)
Supplement: Supplementary file 2 — Additional file 2. Identified factors and factor loadings. Tables with identified factors and factor loadings per item of the extended MMD-HP and EDMCQ. [file 12910_2021_641_MOESM2_ESM.docx]

**ADDITIONAL FILE 2: identified factors and factor loadings**

Table 1. Identified factors and factor loadings per item of the Extended Measure of Moral Distress – Healthcare Professionals (MMD-HP) questionnaire.

| Items per factor | Factor loading |
| --- | --- |
| Factor 1. Suboptimal patientcare due to organizational restrictions |  |
| 13. Be required to work with other healthcare team members who are less experienced than patient care requires. | 0.873 |
| 16. Be required to care for more patients than I can safely care for. | 0.744 |
| 9. Watch patient care suffer because of a lack of provider continuity. | 0.507 |
| 14. Witness low quality of patient care due to poor team communication. | 0.496 |
| 28. Working with other healthcare team members who I don’t know well. | 0.472 |
| 17. Experience compromised patient care due to lack of resources/equipment/bed capacity. | 0.468 |
| 4. Be unable to provide optimal care due to pressures from administrators or insurers to reduce costs. | 0.312 |
| Factor 2. Inadequate emotional support for patients and their families |  |
| 30. Be unable to provide optimal emotional support to anxious and distressed patients/family members | 0.719 |
| 29. Be unable to give patients/family members the opportunity to have a dignified farewell. | -0.682 |
| 35. Providing care to patients of whom the course of the disease and proper treatment is unclear. | -0.517 |
| 34. Be unable to provide family/patient with consistent information, for example due to lack of time or communication facilities. | -0.409 |
| Factor 3. Fear of contamination |  |
| 31. Feeling obligated to provide care to patients where my own health is at risk. | 0.923 |
| 32. Feeling obligated to provide care to patients where the health of my loved ones is at risk. | 0.834 |
| 33. Feeling unsafe due to a limited stock of protective equipment, such as mouth masks, gowns, safety glasses, aprons, gloves and/or disinfectants. | 0.486 |
| Factor 4. Collaboration with patients and their families |  |
| 2. Follow the family’s insistence to continue aggressive treatment even though I believe it is not in the best interest of the patient. | 0.566 |
| 1. Witness healthcare providers giving “false hope” to a patient or family | 0.411 |
| 26. Participate on a team that gives inconsistent messages to a patient/family. | 0.405 |
| 22. Be required to work with abusive patients/family members who are compromising quality of care. | 0.389 |
| Factor 5. Culture of fear and hierarchy |  |
| 21. Feel unsafe/bullied amongst my own colleagues. | 0.705 |
| 25. Work within power hierarchies in teams, units, and my institution that compromise patient care. | 0.520 |
| 6. Be pressured to avoid taking action when I learn that a physician, nurse, or other team colleague has made a medical error and does not report it. | 0.485 |
| 11. Witness a violation of a standard of practice or a code of ethics and not feel sufficiently supported to report the violation. | 0.458 |
| 15. Feel pressured to ignore situations in which patients have not been given adequate information to ensure informed consent. | 0.390 |
| 12. Participate in care that I do not agree with but do so because of fears of litigation. | 0.349 |
| Factor 6. Administrative burden |  |
| 23. Feel required to overemphasize tasks and productivity or quality measures at the expense of patient care. | 0.510 |
| 19. Have excessive documentation requirements that compromise patient care. | 0.406 |
| Factor 7. Disproportionality and aimlessness |  |
| 5. Continue to provide aggressive treatment for a person who is most likely to die regardless of this treatment when no one will make a decision to withdraw it. | 0.547 |
| 3. Feel pressured to order or carry out orders for what I consider to be unnecessary or inappropriate tests and treatments. | 0.540 |
| 8. Participate in care that causes unnecessary suffering or does not adequately relieve pain or symptoms. | 0.417 |

Table 2. Identified factors and factor loadings per item of the Extended Ethical Decision-Making Climate Questionnaire (EDMCQ).

| Items per factor | Factor loading |
| --- | --- |
| Factor 1. Practice and culture of ethical awareness and support |  |
| … experiences and concerns are shared with each other | 0.857 |
| … I can vent my heart | 0.805 |
| … sufficient attention is paid to the impact of the situation on each other | 0.760 |
| … Colleagues regularly inquire about my concerns and needs. | 0.683 |
| … Differences in personal or family circumstances of colleagues are respected | 0.610 |
| … Differences in culture and religion of colleagues are respected. | 0.592 |
| ... my colleagues understand my ideas/feelings about difficult end-of-life decisions. | 0.482 |
| ... different opinions and values are tolerated. | 0.421 |
| ... we talk about moral and ethical problems. | 0.413 |
| ... the culture in the department makes it easy to learn from the mistakes of others. | 0.340 |
| … I know where I can go for professional psychosocial support. | 0.333 |
| Factor 2. Self-reflective and empowering leadership by physicians |  |
| … Physicians in charge are well aware of their own emotions and attitudes | 0.770 |
| … Physicians in charge dare to show their vulnerability | 0.744 |
| … Physicians in charge are well aware of their role model function. | 0.731 |
| … Physicians in charge treat all team members as their equals | 0.667 |
| … Physicians in charge encourage initiative in the team members. | 0.628 |
| … Physicians in charge trust the team members to exercise good judgment. | 0.619 |
| … Physicians in charge help team members settle their differences. | 0.597 |
| … Physicians in charge permit the team members to use their own judgment in solving problems. | 0.540 |
| … Physicians in charge are well aware of their own emotions and attitudes | 0.770 |
| Factor 3. Culture of not avoiding end-of-life decisions |  |
| ⱡ … Patients with little chance of recovery do not frequently occupy an ICU bed which other patients would benefit more from | 0.821 |
| ⱡ … Patients with little chance of recovery are not frequently admitted. | 0.796 |
| ⱡ … EOL decisions are not frequently postponed. | 0.680 |
| ⱡ … Death is not perceived as a treatment failure, so decisions to withdraw or withhold therapy are seldom postponed. | 0.509 |
| Factor 4. Practice and culture of open interdisciplinary reflection/discussion |  |
| … We regularly reflect on the quality of care provided from the various points of view of the staff. | -0.779 |
| … There is regular structured and formal dialogue between the various disciplines within the team to discuss patient care. | -0.597 |
| … There are regular opportunities for open informal dialogue between healthcare providers. | -0.482 |
| … The teams are well coordinated/managed. | -0.465 |
| … Discussions about patients lead to greater understanding and agreements. | -0.448 |
| … There is an open and constructive culture in the department such that criticism can be easily expressed. | -0.444 |
| … There is a structured, formal debriefing after difficult patient care situation | -0.334 |
| Factor 5. Active involvement of nurses in end-of-life care and decision making |  |
| … Nurses are involved in end-of-life decision-making. | -0.794 |
| … Nurses and physicians collaborate well with one another during end-of-life situations. | -0.706 |
| … Nurses are present during the communication of end-of-life information to the family. | -0.692 |
| Factor 6. Relaxation after or during work |  |
| .… I have enough sleep to be rested at work. | 0.779 |
| .… I have enough time for relaxation next to work. | 0.731 |
| .… I can adequately focus on my work during my shifts. | 0.552 |
| .… I have enough breaks to eat, drink and relax. | 0.548 |
| … there is calmness, control and overview in the department. | 0.520 |
| Factor 7. Culture of mutual respect within the interdisciplinary team |  |
| … I am always regarded and addressed by everyone in the team as a full-fledged team member. | 0.784 |
| … Team members from another discipline respect my work. | 0.671 |
| … I have confidence in the professional competence of my team members. | 0.326 |
| Factor 8. Active decision making by physicians |  |
| … Physicians in charge take full charge when emergencies arise. | 0.570 |

Every item is scored on a 5-point Likert scale from 1 (strongly disagree) to 5 (strongly agree).
ⱡ = items are reverse scored
