# Supplementary material for: Moral distress and ethical climate in intensive care medicine during COVID-19: a nationwide study
Source: BMC Med Ethics. 2021 Jun 17;22:73. doi: 10.1186/s12910-021-00641-3 (PMC8211309; doi:10.1186/s12910-021-00641-3)
Supplement: Supplementary file 3 — Additional file 3. Exploratory factor analysis and internal consistency. Tables with identified factors, number of items, percentage of variance, mean scale score and Cronbach’s alpha explained by each factor of the extended MMD-HP and EDMCQ. [file 12910_2021_641_MOESM3_ESM.docx]

**ADDITIONAL FILE 3: Exploratory factor analysis and internal consistency**

Table 1. Extended MMD-HP factors identified, number of items, percentage of variance, mean scale score and Cronbach’s alpha explained by each factor (no. of cases = 504)

| Factor no. | Factor description | Percentage of variance explained by factor | Number of items per factor | Mean scale score | Cronbach α^a^ |
| --- | --- | --- | --- | --- | --- |
| 1 | Suboptimal patientcare due to organizational restrictions | 30.6 | 7 | 4.40 | 0.86 |
| 2 | Inadequate emotional support for patients and their families | 6.0 | 4 | 5.53 | 0.81 |
| 3 | Fear of contamination | 4.5 | 3 | 3.92 | 0.84 |
| 4 | Collaboration with patients and their families | 2.6 | 4 | 1.38 | 0.67 |
| 5 | Culture of fear and hierarchy | 2.3 | 6 | 0.80 | 0.77 |
| 6 | Administrative burden | 1.6 | 2 | 2.73 | 0.67 |
| 7 | Disproportionality and aimlessness | 1.4 | 3 | 2.36 | 0.76 |

^a^Cronbach’s alpha ranges from 0 to 1: the higher the coefficient, the more consistent the scale.

Table 2. Extended EDMCQ factors identified, number of items, percentage of variance, mean scale score and Cronbach alpha explained by each factor (no. of cases = 488)

| Factor no. | Factor description | Percentage of variance explained by factor | Number of items per factor | Mean scale score | Cronbach α^a^ |
| --- | --- | --- | --- | --- | --- |
| 1 | Practice and culture of ethical awareness and support | 30.7 | 11 | 4.31 | 0.91 |
| 2 | Self-reflective and empowering leadership by physicians | 6.5 | 8 | 3.51 | 0.89 |
| 3 | Culture of not avoiding end-of-life decisions | 4.2 | 4 | 3.40 | 0.79 |
| 4 | Practice and culture of open interdisciplinary reflection/discussion | 3.5 | 7 | 3.80 | 0.86 |
| 5 | Active involvement of nurses in end-of-life care and decision making | 3.0 | 3 | 3.76 | 0.84 |
| 6 | Relaxation after or during work | 2.6 | 5 | 3.86 | 0.80 |
| 7 | Culture of mutual respect within the interdisciplinary team | 2.0 | 3 | 4.30 | 0.77 |
| 8 | Active decision making by physicians | 1.3 | 1 | 4.24 |  |

^a^Cronbach’s alpha ranges from 0 to 1: the higher the coefficient, the more consistent the scale. 0.7 is an acceptable internal consistency coefficient, but lower thresholds are sometimes used.
