# Supplementary material for: Moral distress and ethical climate in intensive care medicine during COVID-19: a nationwide study
Source: BMC Med Ethics. 2021 Jun 17;22:73. doi: 10.1186/s12910-021-00641-3 (PMC8211309; doi:10.1186/s12910-021-00641-3)
Supplement: Supplementary file 4 — Additional file 4. Highest and lowest scoring items per factor. Overview of items with the highest and lowest scores per factor of the extended MMD-HP for ICU nurses, intensivists and supporting staff. [file 12910_2021_641_MOESM4_ESM.docx]

**ADDITIONAL FILE 4: highest and lowest scoring items per factor**

Table 1. Overview of items with the highest and lowest scores per factor of the extended MMD-HP for ICU nurses (n = 355)

| Factor no. | Factor description | Item no. | Item | Mean item score | Standard deviation |
| --- | --- | --- | --- | --- | --- |
| 1 | Suboptimal patientcare due to organisational restrictions | 13 | Be required to work with other healthcare team members who are less experienced than patient care requires. | 6.57 | 4.95 |
|  |  | 4 | Be unable to provide optimal care due to pressures from administrators or insurers to reduce costs. | 3.05 | 4.4 |
| 2 | Inadequate emotional support for patients and their families | 30 | Be unable to provide optimal emotional support to anxious and distressed patients/family members. | 7.69 | 5.31 |
|  |  | 34 | Be unable to provide family/patient with consistent information, for example due to lack of time or communication facilities. | 3.84 | 4.51 |
| 3 | Fear of contamination | 32 | Feeling obligated to provide care to patients where the health of my loved ones is at risk. | 4.30 | 5.10 |
|  |  | 33 | Feeling unsafe due to a limited stock of protective equipment, such as mouth masks, gowns, safety glasses, aprons, gloves and/or disinfectants. | 3.31 | 4.63 |
| 4 | Collaboration with patients and their families | 2 | Follow the family’s insistence to continue aggressive treatment even though I believe it is not in the best interest of the patient. | 2.26 | 2.96 |
|  |  | 22 | Be required to work with abusive patients/family members who are compromising quality of care. | 1.03 | 2.24 |
| 5 | Culture of fear and hierarchy | 25 | Work within power hierarchies in teams, units, and my institution that compromise patient care. | 1.43 | 2.84 |
|  |  | 21 | Feel unsafe/bullied amongst my own colleagues. | 0.57 | 2.08 |
| 6 | Administrative burden | 19 | Have excessive documentation requirements that compromise patient care. | 3.66 | 4.30 |
|  |  | 23 | Feel required to overemphasize tasks and productivity or quality measures at the expense of patient care. | 2.27 | 3.79 |
| 7 | Disproportional and aimless care | 5 | Continue to provide aggressive treatment for a person who is most likely to die regardless of this treatment when no one decides to withdraw it. | 3.84 | 4.03 |
|  |  | 3 | Feel pressured to order or carry out orders for what I consider unnecessary or inappropriate tests and treatments. | 2.07 | 2.83 |

Table 2. Overview of items with the highest and lowest scores per factor of the extended MMD-HP for intensivists (n=41)

| Factor no. | Factor description | Item no. | Item | Mean item score | Standard deviation |
| --- | --- | --- | --- | --- | --- |
| 1 | Suboptimal patientcare due to organizational restrictions | 17 | Experience compromised patient care due to a lack of resources/equipment/bed capacity. | 6.10 | 5.25 |
|  |  | 4 | Be unable to provide optimal care due to pressures from administrators or insurers to reduce costs. | 2.10 | 3.06 |
| 2 | Inadequate emotional support for patients and their families | 29 | Be unable to allow patients/family members to have a dignified farewell. | 8.02 | 4.87 |
|  |  | 34 | Be unable to provide family/patient with consistent information, for example due to lack of time or communication facilities. | 4.10 | 3.60 |
| 3 | Fear of contamination | 32 | Feeling obligated to provide care to patients, where the health of my loved ones is at risk. | 4.22 | 4.87 |
|  |  | 33 | Feeling unsafe due to a limited stock of protective equipment, such as mouth masks, gowns, safety glasses, aprons, gloves and/or disinfectants. | 3.24 | 4.71 |
| 4 | Collaboration with patients and their families | 2 | Follow the family’s insistence to continue aggressive treatment even though I believe it is not in the best interest of the patient. | 2.17 | 2.05 |
|  |  | 22 | Be required to work with abusive patients/family members who are compromising quality of care. | 0.85 | 1.31 |
| 5 | Culture of fear and hierarchy | 25 | Work within power hierarchies in teams, units, and my institution that compromise patient care. | 2.85 | 3.82 |
|  |  | 11 | Witness a violation of a standard of practice or a code of ethics and not feel sufficiently supported to report the violation. | 0.51 | 0.98 |
| 6 | Administrative burden | 19 | Have excessive documentation requirements that compromise patient care. | 3.51 | 3.78 |
|  |  | 23 | Feel required to overemphasize tasks and productivity or quality measures at the expense of patient care | 1.34 | 2.63 |
| 7 | Disproportionality and aimlessness | 5 | Continue to provide aggressive treatment for a person who is most likely to die regardless of this treatment when no one will make a decision to withdraw it. | 2.39 | 2.72 |
|  |  | 8 | Participate in care that causes unnecessary suffering or does not adequately relieve pain or symptoms | 1.39 | 1.83 |

Table 3. Overview of items with the highest and lowest scores per factor of the extended MMD-HP for supporting staff (n=108)

| Factor no. | Factor description | Item no. | Item | Mean item score | Standard deviation |
| --- | --- | --- | --- | --- | --- |
| 1 | Suboptimal patientcare due to organizational restrictions | 28 | Working with other healthcare team members who I do not know well. | 4.81 | 4.28 |
|  |  | 4 | Be unable to provide optimal care due to pressures from administrators or insurers to reduce costs. | 1.50 | 2.94 |
| 2 | Inadequate emotional support for patients and their families | 29 | Be unable to allow patients/family members to have a dignified farewell. | 6.55 | 5.25 |
|  |  | 34 | Be unable to provide family/patient with consistent information, for example due to lack of time or communication facilities. | 2.04 | 3.43 |
| 3 | Fear of contamination | 32 | Feeling obligated to provide care to patients where the health of my loved ones is at risk. | 4.37 | 4.86 |
|  |  | 33 | Feeling unsafe due to a limited stock of protective equipment, such as mouth masks, gowns, safety glasses, aprons, gloves and/or disinfectants | 3.19 | 4.56 |
| 4 | Collaboration with patients and their families | 2 | Follow the family’s insistence to continue aggressive treatment even though I believe it is not in the best interest of the patient. | 1.32 | 2.24 |
|  |  | 22 | Be required to work with abusive patients/family members who are compromising quality of care. | 0.28 | 1.26 |
| 5 | Culture of fear and hierarchy | 25 | Work within power hierarchies in teams, units, and my institution that compromise patient care. | 1.23 | 2.43 |
|  |  | 6 | Be pressured to avoid taking action when I learn that a physician, nurse, or other team colleague has made a medical error and does not report it. | 0.18 | 0.64 |
| 6 | Administrative burden | 19 | Have excessive documentation requirements that compromise patient care. | 2.82 | 4.03 |
|  |  | 23 | Feel required to overemphasize tasks and productivity or quality measures at the expense of patient care. | 1.32 | 2.92 |
| 7 | Disproportionality and aimlessness | 5 | Continue to provide aggressive treatment for a person who is most likely to die regardless of this treatment when no one will make a decision to withdraw it. | 2.18 | 2.94 |
|  |  | 3 | Feel pressured to order or carry out orders for what I consider to be unnecessary or inappropriate tests and treatments. | 1.08 | 1.87 |
