# Supplementary material for: Moral distress and ethical climate in intensive care medicine during COVID-19: a nationwide study
Source: BMC Med Ethics. 2021 Jun 17;22:73. doi: 10.1186/s12910-021-00641-3 (PMC8211309; doi:10.1186/s12910-021-00641-3)
Supplement: Supplementary file 5 — Additional file 5. Response-percentages additional questions. Response-percentages of each professional group per additional question. [file 12910_2021_641_MOESM5_ESM.docx]

**ADDITIONAL FILE 5: Response-percentages additional questions**

Table 1. Response-percentages of each professional group per additional question

|  | Nurse | Intensivist | Supporting staff | p-value |
| --- | --- | --- | --- | --- |
| Have you ever left or considered leaving a clinical position due to moral distress? |  |  |  | <0.05 |
| No, I have never considered leaving or left a position | 269 (75.8%) | 32 (78.0%) | 86 (79.6%) |  |
| Yes, I considered leaving but did not leave | 71 (20.0%) | 7 (17.1%) | 9 (8.3%) |  |
| Yes, I left a position | 15 (4.2%) | 2 (4.9%) | 13 (12.0%) |  |
| Are you considering leaving your position now due to moral distress? |  |  |  | 0.975 |
| Yes | 25 (7.0%) | 3 (7.3%) | 7 (6.5%) |  |
| No | 330 (93.0%) | 38 (92.7%) | 101 (93.5%) |  |
| Is attention paid to moral distress in your hospital? |  |  |  | 0.161 |
| Yes | 334 (94.1%) | 36 (87.8%) | 100 (92.6%) |  |
| No | 7 (2.0%) | 4 (9.8%) | 2 (1.9%) |  |
| I don’t know | 14 (3.9%) | 1 (2.4%) | 6 (5.6%) |  |
| Do you think enough attention is paid to reduce moral distress? |  |  |  | 0.547 |
| Yes | 284 (80.5%) | 34 (82.9%) | 91 (85.0%) |  |
| No | 69 (19.5%) | 7 (17.1%) | 16 (15.0%) |  |
| Have you used or considered using any professional psychosocial help during COVID-19 due to moral distress? |  |  |  | 0.687 |
| No, I have never considered using or used professional psychosocial help | 264 (77.6%) | 28 (70.0%) | 75 (75.0%) |  |
| Yes, I considered using it but did not use it | 46 (13.5%) | 6 (15.0%) | 13 (13.0%) |  |
| Yes, I used professional psychosocial help | 30 (8.8%) | 6 (15.0%) | 12 (12.0%) |  |
| Are you considering using professional psychosocial help now? |  |  |  | <0.05 |
| Yes | 15 (4.4%) | 6 (15.0%) | 9(9.0%) |  |
| No | 325 (95.6%) | 34 (85.0%) | 91 (91.0%) |  |
