# Supplementary material for: Moral distress and ethical climate in intensive care medicine during COVID-19: a nationwide study
Source: BMC Med Ethics. 2021 Jun 17;22:73. doi: 10.1186/s12910-021-00641-3 (PMC8211309; doi:10.1186/s12910-021-00641-3)
Supplement: Supplementary file 6 — Additional file 6. Lowest ranking ethical climate items. Overview of the lowest ranking ethical climate items in the extended EDMCQ per profession. [file 12910_2021_641_MOESM6_ESM.docx]

**ADDITIONAL FILE 6: Lowest ranking ethical climate items**

Table 1. Lowest ranking ethical climate items in the extended EMDCQ

|  | Nurses (n=345) | | Intensivists (n=40) | | Supporting staff (n=103) | |  |
| --- | --- | --- | --- | --- | --- | --- | --- |
| Item | Mean (SD) | Rank | Mean (SD) | Rank | Mean (SD) | Rank | p-value |
| Physicians in charge dare to show their vulnerability | 2.99 (0.99) | 1 | 3.40 (0.81) | 2 | 3.16 (0.85) | 3 | <0.05 |
| Patients with little chance of recovery are not frequently admitted | 3.00 (0.15) | 2 | 3.25 (1.10) | 1 | 3.01 (0.91) | 1 | 0.395 |
| End-of-life decisions are not frequently postponed | 3.21 (1.10) | 3 | 3.70 (1.07) | 8 | 3.15 (0.88) | 2 | <0.05 |
| There is a structured, formal debriefing after a difficult patient care situation | 3.23 (0.20) | 4 | 3.63 (1.08) | 5 | 3.37 (1.14) | 6 | 0.107 |
| Physicians in charge are well aware of their own emotions and attitudes | 3.26 (0.87) | 5 | 3.50 (0.75) | 4 | 3.28 (0.90) | 4 | 0.251 |
| Patients with little chance of recovery do not frequently occupy an ICU bed which other patients would benefit more from | 3.29 (1.18) | 6 | 3.70 (1.02) | 9 | 3.39 (0.91) | 7 | 0.077 |
| Physicians in charge help team members settle their differences | 3.30 (1.02) | 7 | 3.43 (0.87) | 3 | 3.33 (0.90) | 5 | 0.734 |
| We regularly reflect on the quality of care provided from the various points of view of the staff | 3.46 (1.26) | 8 | 3.70 (1.07) | 7 | 3.61 (1.10) | 11 | 0.318 |
| Physicians in charge are well aware of their role model function | 3.50 (0.84) | 9 | 3.65 (0.66) | 6 | 3.57 (0.76) | 8 | 0.433 |
