# Supplementary material for: Moral distress and ethical climate in intensive care medicine during COVID-19: a nationwide study
Source: BMC Med Ethics. 2021 Jun 17;22:73. doi: 10.1186/s12910-021-00641-3 (PMC8211309; doi:10.1186/s12910-021-00641-3)
Supplement: Supplementary file 7 — Additional file 7. Most morally distressing items compared with 2019. Comparison of the original MMD-HP for the most morally distressing items per profession during COVID-19 and the historical control group one year before COVID-19. [file 12910_2021_641_MOESM7_ESM.docx]

**ADDITIONAL FILE 7: Most morally distressing items compared with 2019**

Table 1. Highest ranking moral distress situations original MMD-HP compared with 2019 for ICU nurses

|  |  | COVID-19 | | 2019 | |  |
| --- | --- | --- | --- | --- | --- | --- |
| Item no. | Item | Mean (SD) | Rank | Mean (SD) | Rank | p-value |
| 13 | Be required to work with other healthcare team members who are less experienced than patient care requires. | 6.57 (4.94) | 1 | 3.32 (3.6) | 11 | <0.001 |
| 16 | Be required to care for more patients than I can safely care for. | 6.22 (5.14) | 2 | 4.29 (4.09) | 8 | <0.001 |
| 17 | Experience compromised patient care due to lack of resources, equipment or bed capacity. | 5.22 (4.97) | 3 | 4.58 (4.36) | 6 | <0.05 |
| 9 | Watch patient care suffer because of a lack of provider continuity. | 5.00 (4,65) | 4 | 4.75 (4.37) | 5 | 0.42 |
| 5 | Continue to provide aggressive treatment for a person who is most likely to die regardless of this treatment when no one will make a decision to withdraw it. | 3.84 (4.03) | 5 | 5.67 (4.07) | 2 | <0.001 |

Table 2. Highest ranking moral distress situations original MMD-HP compared with 2019 for intensivists

|  |  | COVID-19 | | 2019 | |  |
| --- | --- | --- | --- | --- | --- | --- |
| Item no. | Item | Mean (SD) | Rank | Mean (SD) | Rank | p-value |
| 17 | Experience compromised patient care due to lack of resources, equipment or bed capacity. | 6.10 (5.25) | 1 | 5.23 (4.58) | 1 | 0.32 |
| 9 | Watch patient care suffer because of a lack of provider continuity. | 3.823 (3.67) | 2 | 5.04 (3.75) | 2 | <0.05 |
| 18 | Experience lack of administrative action or support for a problem that is compromising patient care. | 3.80 (4.45) | 3 | 4.52 (4.20) | 3 | 0.36 |
| 19 | Have excessive documentation requirements that compromise patient care. | 3.51 (3.77) | 4 | 4.31 (4.29) | 4 | 0.29 |
| 5 | Be required to work with other healthcare team members who are less experienced than care requires. | 3.32 (3.44) | 5 | 3.36 (3.01) | 9 | 0.94 |
